# Supplementary material for: Multiple Transitions between Y Chromosome and Autosome in Tago’s Brown Frog Species Complex
Source: Genes (Basel). 2024 Feb 26;15(3):300. doi: 10.3390/genes15030300 (PMC10969965; doi:10.3390/genes15030300)
Supplement: Supplementary file 1 [file genes-15-00300-s001.zip › Figures S (pdf+ligend).pdf]

Supplemental information (figures) for:

Multiple transitions between Y chromosome and autosome in Tago's brown frog species complex

Ikuo Miura, Foyez Shams, Jun-ichi Ohki, Yasuo Aonishi, Masataka Tagami, Hiroyuki Fujita Chiao Kuwana, Chiyo Nanba, Kosuke Hayashi, Ryoko Yamaji, Takanori Matsuo, Mitsuaki Ogata, Shuuji Mawaribuchi, Norio Shimizu and Tariq Ezaz

Figures S1 – S5

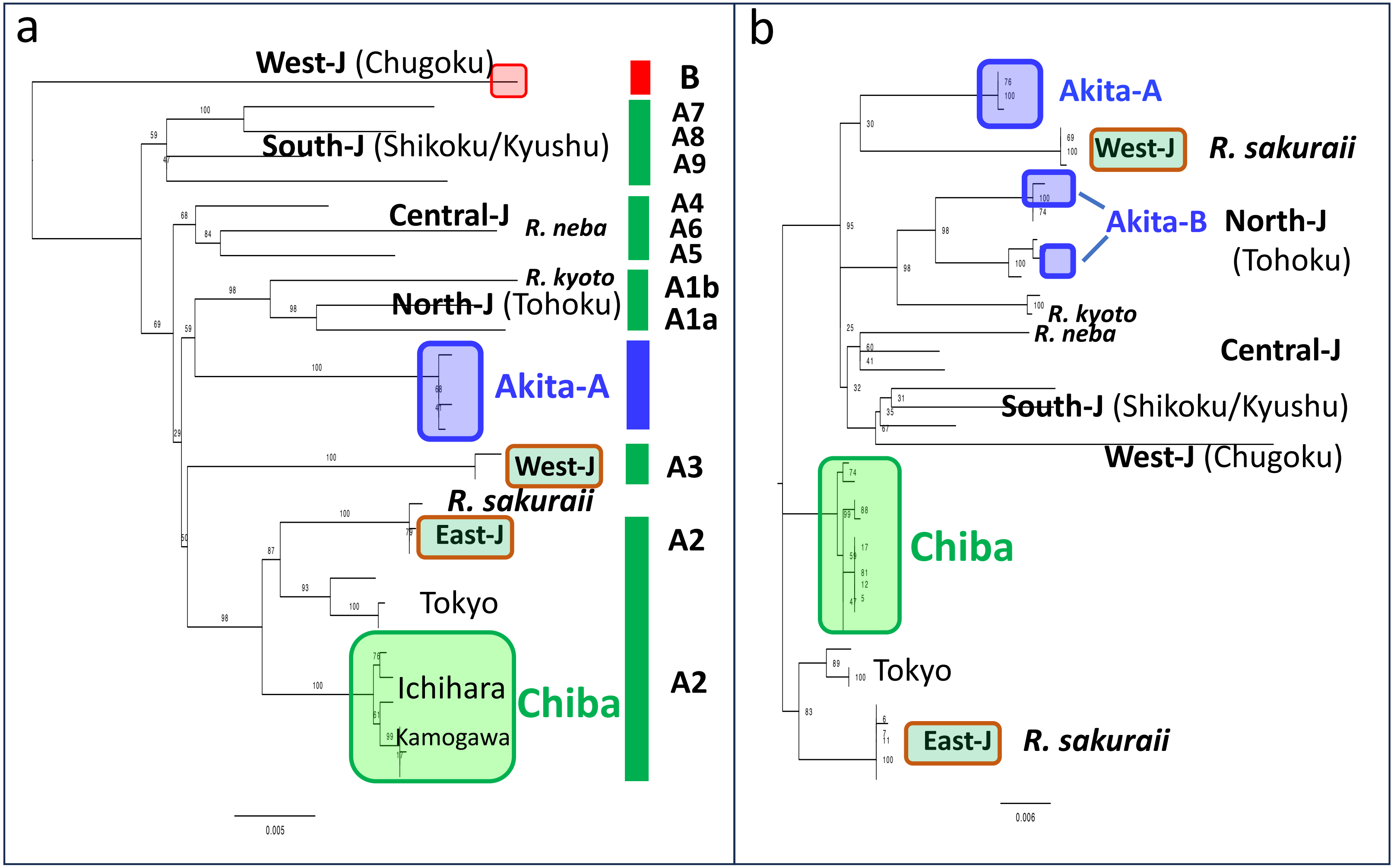

Figure S1. Maximum Likelihood trees of mitochondrial genes (ND1 and 16S rRNA, 2.6 kbp, in a and those, 1.6 kbp, in b. Chiba and Akita populations of *Rana tagoi* are circled in green and blue, respectively. The green, blue, and red boxes in A indicate the genetic groups defined by Eto et al. (2012)[15].

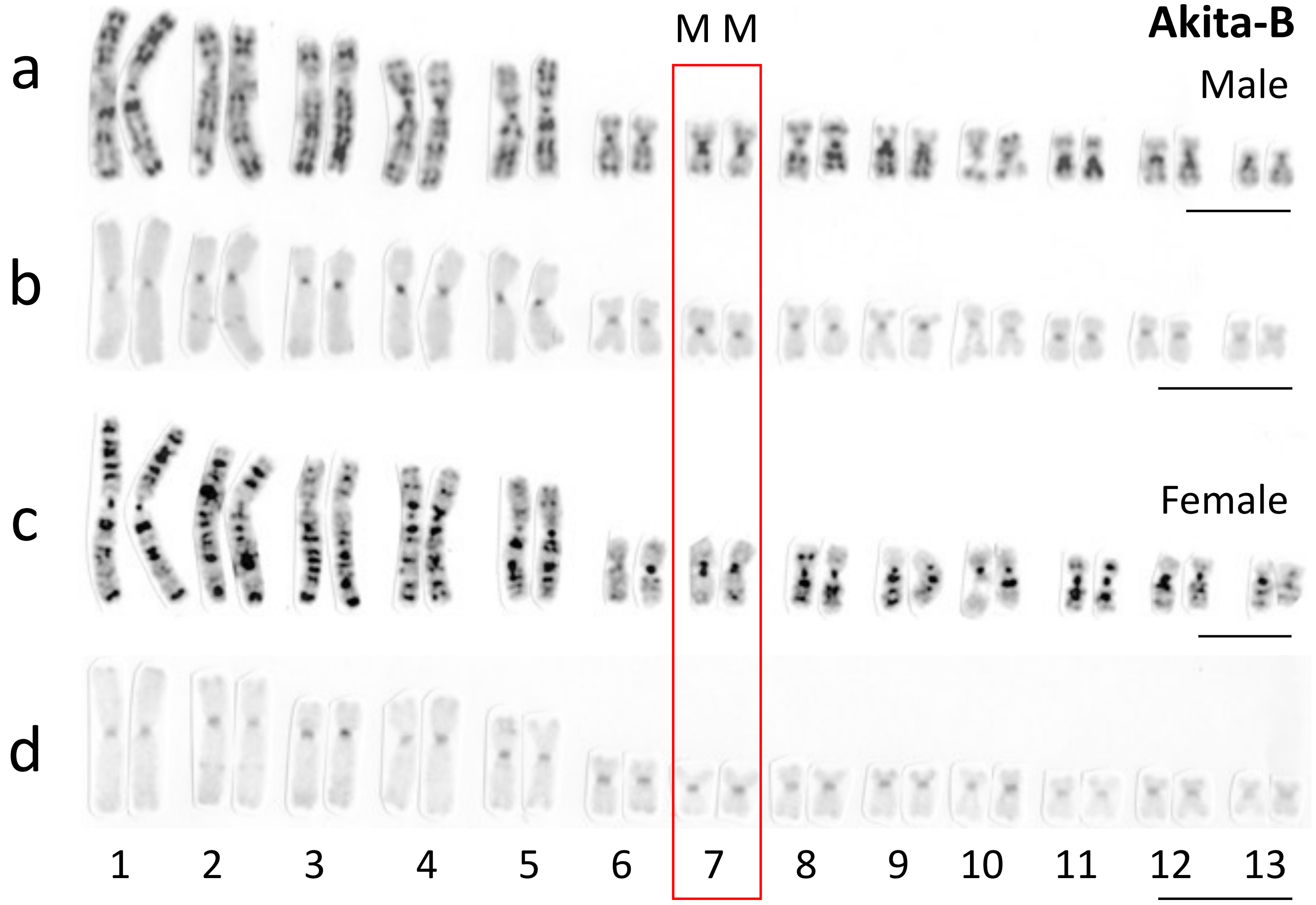

Figure S2. Karyotypes of *Rana tagoi* from the Akita population (Higashi-Naruse B) in North Japan. Late replication banded (a, c) and C-banded chromosomes (b, d). Chromosome 7 (boxed) is metacentric and homomorphic in both the sexes. Bar, 10 μm.

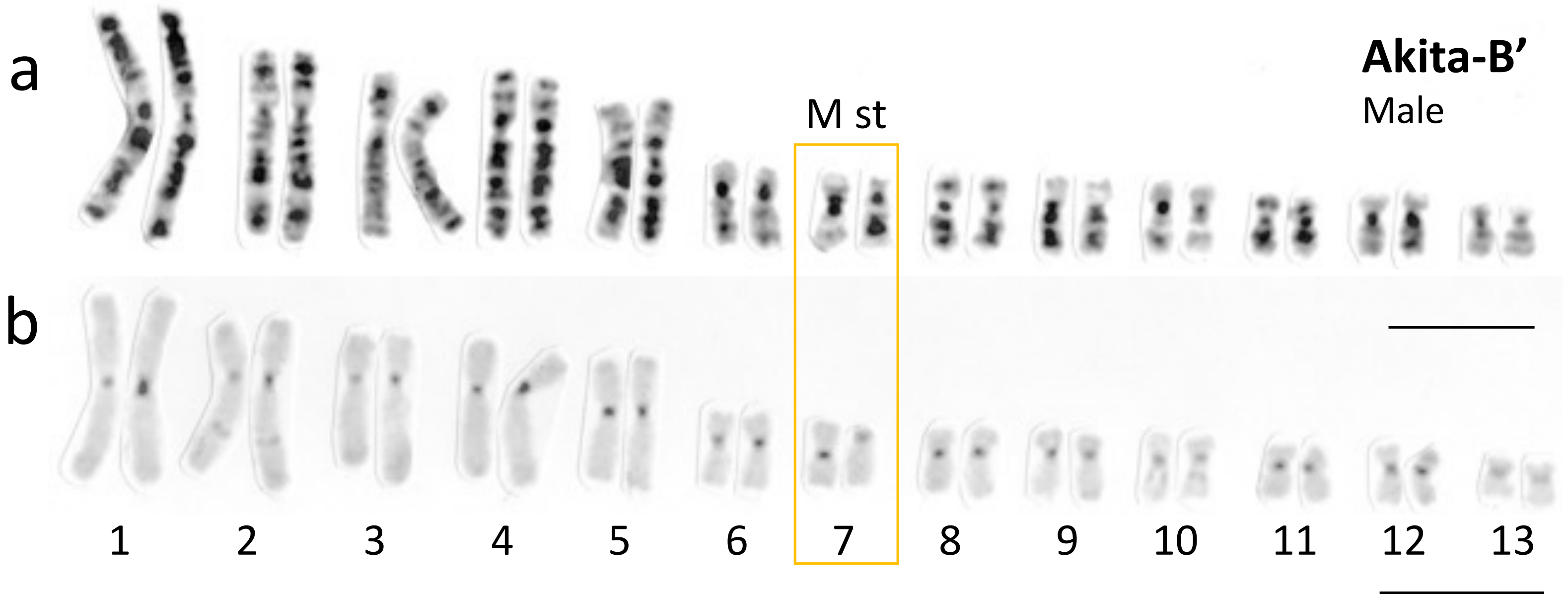

Figure S3. Karyotypes of *Rana tagoi* from the Akita population (Higashi-Naruse B’) in North Japan. Late replication-banded (a) and C-banded (b) chromosomes. Chromosome 7 (boxed) is heteromorphic, metacentric, and subtelocentric in males. Bar, 10 μm.

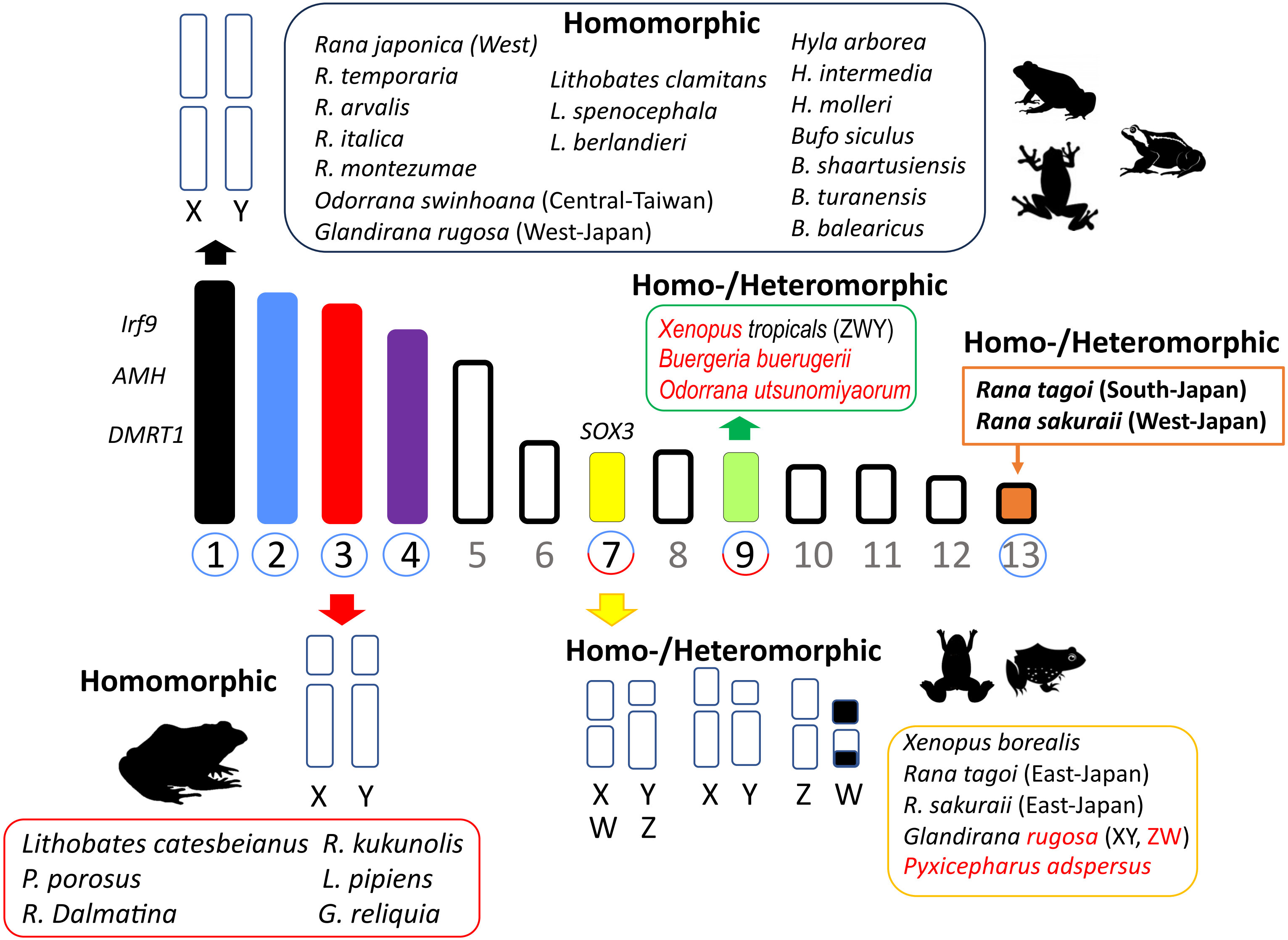

Figure S4. Seven members of the potential sex chromosomes in frogs. Chromosomes 1, 2, 3, 4, 7, 9, and 13 (2n = 26) are the sex chromosomes in the listed species (species bearing sex chromosomes 2 or 4 are not listed here; see Miura, 2017[2]). Sex chromosome 13 has been added in this study. Species with XX-XY and ZZ-ZW types of sex determination are shown in black and red, respectively.
